# SUBTYPE-SPECIFIC OPTIMAL CUT-OFF VALUES OF THE BERG BALANCE SCALE FOR PREDICTING INDEPENDENT WALKING IN INPATIENT STROKE REHABILITATION: A MULTICENTRE COHORT STUDY

**DOI:** 10.2340/jrm.v58.45663

**Published:** 2026-07-08

**Authors:** Jeong-Soo KIM, In-Geon HWANG, Eu-Jeong KO

**Affiliations:** 1 Department of Physical Therapy, College of Health Science, Yonsei University, Wonju; 2 Department of Mirae Rehabilitation Research, Seoul Rehabilitation Hospital, Seoul; 3 Department of Recovery Rehabilitation Medicine, Seoul Rehabilitation Hospital, Seoul, Republic of Korea

**Keywords:** balance, Berg Balance Scale, gait, prediction, rehabilitation, stroke

## Abstract

**Objective:**

To determine whether admission Berg Balance Scale score independently predicts independent walking on discharge after adjustment for major confounders, and to derive subtype-specific optimal cut-off values for ischaemic and haemorrhagic stroke.

**Design:**

Multicentre retrospective cohort study.

**Subjects/Patients:**

A total of 565 stroke patients (316 ischaemic, 249 haemorrhagic) admitted to 3 inpatient rehabilitation centres in the Republic of Korea.

**Methods:**

Multivariable logistic regression was used to evaluate the independent predictive value of the Berg Balance Scale. Optimal cut-off values were derived using receiver operating characteristic curve analysis and the Youden index. Bootstrap internal validation, calibration analysis, and decision curve analysis were performed.

**Results:**

Admission Berg Balance Scale was a significant independent predictor of independent walking (adjusted odds ratio 1.053, 95% confidence interval 1.030–1.076). The difference in discriminative ability between the Berg Balance Scale only and multivariable models was not statistically significant (*p* = 0.097). The overall optimal cut-off was 24 points; subtype-specific cut-offs were 33 for ischaemic and 12 for haemorrhagic stroke.

**Conclusion:**

The Berg Balance Scale has different optimal cut-off values by stroke subtype and, as a standalone assessment, maintains discriminative ability equivalent to a multivariable model, providing clinical evidence for subtype-specific precision rehabilitation strategies.

## LAY ABSTRACT

Whether a person can walk independently again after a stroke is of paramount concern to patients and their families. This study examined 565 stroke patients admitted to 3 hospitals in Korea and investigated whether a simple balance test performed at the start of rehabilitation could predict the ability to walk independently on discharge. The results showed that higher balance scores were associated with a greater likelihood of achieving independent walking, and a balance test alone predicted outcomes as well as a model combining multiple clinical measures. Notably, the balance score needed to predict walking was markedly different between strokes caused by a blocked blood vessel (33 points) and those caused by bleeding (12 points). These findings can inform individualized goal-setting and rehabilitation planning tailored to the type of stroke.

Stroke is a leading cause of long-term disability in adults, and a substantial proportion of survivors experience gait impairment (1). The Copenhagen Stroke Study reported that approximately 63% of patients in the early post-stroke phase were either non-ambulatory or required assistance for walking (2), and recovery of independent walking is consistently identified by patients and caregivers as the highest priority goal of rehabilitation (3). Predicting walking outcomes early during inpatient rehabilitation is clinically important for individualized goal-setting, efficient resource allocation, and evidence-based prognostic communication with patients and families (4).

The Berg Balance Scale (BBS) is a standardized clinical balance assessment comprising 14 items, with established reliability and validity in stroke populations and widespread use in inpatient rehabilitation settings (5, 6). The BBS can be administered in 15–20 min without specialized equipment and has a test–retest intraclass correlation coefficient (ICC) of 0.98 (5). Unlike impairment-level measures such as the Fugl-Meyer Assessment and Trunk Impairment Scale, the BBS evaluates functional balance tasks that are direct prerequisites for walking and maps onto the ICF activity domain (33), and systematic reviews have shown that admission BBS scores are significantly associated with discharge destination, length of stay, and post-stroke motor function (5), and Louie and Eng (7) reported that a BBS cut-off of 29 predicted community ambulation on discharge with an area under the receiver operating characteristic curve (AUC) of 0.88. However, previous studies have predominantly been single-centre with small sample sizes, and studies comparing BBS predictive performance between ischaemic and haemorrhagic stroke subtypes within the same cohort are scarce (4, 8, 9). Studies adjusting simultaneously for stroke severity, cognition, and ambulatory status have been limited (10).

Functional recovery after ischaemic stroke depends primarily on corticospinal tract integrity and neuroplasticity (11), whereas haemorrhagic stroke may involve additional reversible recovery through haematoma absorption and resolution of perihematomal oedema, such that independent walking may be achieved from relatively lower initial balance scores (12, 13, 14, 15). Perna and Temple (16) reported no significant difference in the magnitude of functional recovery between the 2 subtypes, indicating that the evidence remains inconclusive. These pathophysiological differences suggest that the balance threshold required for walking recovery may differ by subtype. The present study aimed to test the hypothesis that the optimal BBS cut-off for predicting independent walking differs between ischaemic and haemorrhagic stroke.

Accordingly, we conducted a multicentre retrospective cohort study of 565 stroke patients across 3 inpatient rehabilitation centres in the Republic of Korea. The primary objective was to verify that admission BBS score is an independent predictor of independent walking (Functional Ambulation Category [FAC] ≥ 4) on discharge after adjustment for age, sex, stroke type, cognitive function (Mini-Mental State Examination; MMSE), and admission ambulatory function (FAC). Secondary objectives included: (*i*) derivation of overall and subtype-specific optimal BBS cut-off values using the Youden index; (*ii*) comparison of AUC between BBS-only and multivariable models using the DeLong test; (*iii*) testing of discriminative homogeneity between subtypes; and (*iv*) evaluation of clinical utility through bootstrap internal validation, calibration analysis, and decision curve analysis (DCA).

## METHODS

### Study design and setting

This was a multicentre retrospective cohort study using data from 3 inpatient rehabilitation centres in the Republic of Korea: 1 acute university hospital (Centre A) and 2 subacute rehabilitation hospitals (Centres B and C). Centres B and C are subacute rehabilitation hospitals with a typical inpatient rehabilitation duration of approximately 90–120 days; the median rehabilitation length of stay observed at these centres (Centre B: 201 days, Centre C: 180 days) exceeded this range because some patients had been transferred from 1 or more other rehabilitation facilities prior to enrolment at the study centre. The participating centres represented the spectrum of stroke rehabilitation delivery, from acute inpatient rehabilitation to long-term subacute rehabilitation. This study was reported in accordance with the Strengthening the Reporting of Observational Studies in Epidemiology (STROBE) guidelines (17) and the Transparent Reporting of a multivariable prediction model for Individual Prognosis or Diagnosis (TRIPOD) guidelines (18).

### Participants

**Fig. 1:**
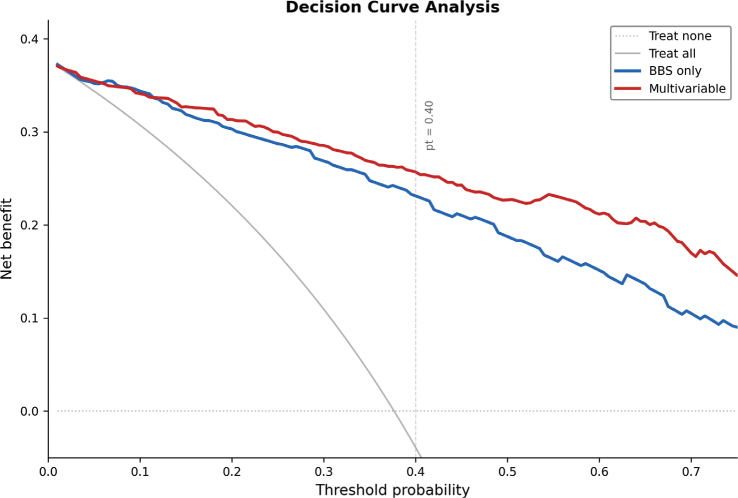
STROBE flow diagram of participant selection.

Patients meeting all of the following criteria were included: (*i*) a diagnosis of ischaemic or haemorrhagic stroke confirmed by computed tomography (CT) or magnetic resonance imaging (MRI); (*ii*) participation in an inpatient rehabilitation programme (outpatient or day-hospital rehabilitation patients were excluded); (*iii*) availability of BBS assessment data on rehabilitation admission (T0); and (*iv*) availability of FAC assessment data on both admission (T0) and discharge (T1). Of the 1,117 patients initially registered, 6 were excluded for non-ischaemic/haemorrhagic stroke type or missing type data, 524 for missing BBS T0, FAC T0, or FAC T1 data, 10 for outpatient rehabilitation, and 4 for missing rehabilitation format data, resulting in 573 provisionally included patients. One centre (*n* = 8) was further excluded because discharge BBS data were entirely missing and the sample size was insufficient for analysis, yielding a final sample of 565 patients (Fig. 1).

### Outcome measures

The primary outcome was independent walking on discharge, defined as FAC ≥ 4. The FAC classifies ambulatory ability on a 0–5 ordinal scale based on the level of human assistance required (23); a score of ≥ 4 indicates the ability to walk independently on level surfaces regardless of the use of assistive devices. Secondary outcomes included discharge Modified Barthel Index (MBI) score and FAC change (discharge score minus admission score).

### Primary predictor

The primary predictor was BBS score on rehabilitation admission. The BBS comprises 14 items assessing static and dynamic balance during functional tasks (19), each scored from 0 to 4, yielding a total score of 0–56. Higher scores indicate better balance ability.

### Covariates

Based on clinical importance and prior literature (4, 20), the following covariates were included: age (years), sex (male/female), stroke type (ischaemic/haemorrhagic), lesion side (right/left/bilateral), body mass index (BMI, kg/m²), comorbidities (hypertension, diabetes mellitus, atrial fibrillation, dyslipidaemia), cognitive function (admission MMSE (21)), admission MBI (22), and admission FAC. NIHSS data were available for only 21.4% (121 patients) of the study population and were therefore not included in the primary analysis model; they were used in a separate sensitivity analysis (see below).

### Statistical analysis

All statistical analyses were performed using R version 4.5.2 (pROC and car packages; R Foundation for Statistical Computing, Vienna, Austria), with a two-sided significance level of *p* < 0.05. Continuous variables were described as mean (SD), and categorical variables as frequencies and percentages. Between-group comparisons (Table I) were performed using the Mann–Whitney *U* test for continuous variables and the chi-square test for categorical variables.

**Table I:** Baseline characteristics of study participants by independent walking status on discharge

Variable	Total (*n* = 565)	Independent (*n* = 214)	Non-independent (*n* = 351)	*p*-value
Age, years	61.3 ± 13.9	57.7 ± 13.8	63.4 ± 13.4	< 0.001
Male, *n* (%)	343 (60.7)	139 (65.0)	204 (58.1)	0.127
BMI, kg/m²	23.3 ± 3.6	24.1 ± 3.6	22.9 ± 3.6	< 0.001
Ischaemic stroke, *n* (%)	316 (55.9)	133 (62.1)	183 (52.1)	**0.025**
Onset to admission, days^†^	42 [23–73]	29 [18–59]	48 [25–78]	< 0.001
Rehabilitation stay, days^†^	74 [44–186]	67 [39–165]	82 [47–195]	**0.027**
BBS on admission	24.2 ± 20.3	41.8 ± 12.8	13.5 ± 16.2	< 0.001
FAC on admission	1.7 ± 1.4	2.9 ± 1.2	0.9 ± 0.9	< 0.001
MMSE on admission	18.3 ± 10.2	22.5 ± 8.0	15.7 ± 10.6	< 0.001
MBI on admission	44.9 ± 37.1	69.9 ± 42.4	29.5 ± 22.4	< 0.001
NIHSS^‡^	7.4 ± 6.1	6.1 ± 5.3	8.4 ± 6.4	**0.034**
Hypertension, *n* (%)	403 (71.3)	143 (66.8)	260 (74.1)	0.080
Diabetes mellitus, *n* (%)	165 (29.2)	54 (25.2)	111 (31.6)	0.117
Atrial fibrillation, *n* (%)	65 (11.5)	20 (9.3)	45 (12.8)	0.261
Dyslipidaemia, *n* (%)	122 (21.6)	35 (16.4)	87 (24.8)	**0.026**

Values are mean ± SD unless otherwise specified.

† Median [interquartile range].

‡ NIHSS available in 122 patients (21.6%).

BBS: Berg Balance Scale; BMI: body mass index; FAC: Functional Ambulation Category; MBI: Modified Barthel Index; MMSE: Mini-Mental State Examination; NIHSS: National Institutes of Health Stroke Scale.

Variables with p < 0.10 in univariable logistic regression were selected as candidates for the multivariable model (Hosmer–Lemeshow recommendation (26)). Sex and stroke type were included by forced entry regardless of univariable *p*-values, based on clinical rationale from prior literature (4, 20). Prior to model building, pairwise Spearman correlations were examined among candidate predictors. Admission MBI was excluded from the primary model due to high collinearity with BBS (ρ = 0.850, *p* < 0.001) and with FAC (ρ = 0.785, *p* < 0.001), which would result in unstable coefficient estimation if retained alongside both BBS and FAC. Admission FAC was retained despite high collinearity with BBS (ρ = 0.856, *p* < 0.001) on clinical grounds, as it represents the most direct antecedent measure of the outcome variable (FAC ≥ 4) and its independent prognostic contribution has been established in prior literature (4, 20). Multivariable logistic regression included BBS as the primary predictor, with adjustment for age, sex, stroke type, admission MMSE, and admission FAC. To account for between-centre heterogeneity in onset-to-admission intervals, the enhanced model included log-transformed onset-to-admission interval [log(Onset_T0+1)] as an additional covariate and was designated as the primary analysis model. Results were expressed as adjusted odds ratios (aOR) with 95% confidence intervals (CI). Model fit was assessed using the McFadden pseudo-R². Missing data in the primary model involved 10 patients (1.8%) due to missing MMSE data (< 5% threshold), and complete case analysis was applied. NIHSS missingness (78.6%, missing not at random pattern) was addressed through separate sensitivity analysis.

The discriminative ability of BBS was assessed using the AUC of the receiver operating characteristic (ROC) curve. The 95% CI for the AUC was computed using 1,000 bootstrap resamples, and the optimal cut-off was determined by the Youden index (sensitivity + specificity – 1). Sensitivity, specificity, positive predictive value (PPV), and negative predictive value (NPV) at multiple cut-off points (BBS 10–45) are presented in Table III. AUC comparison between BBS-only and multivariable models was performed using the DeLong test (24). Subtype-specific AUC comparisons were performed using the same method. Effect heterogeneity by stroke subtype was assessed using a likelihood ratio test with a BBS × stroke type interaction term. BBS predictive performance was also examined by stratification according to admission FAC level.

Internal validity and overfitting were assessed using Harrell’s bootstrap method (1,000 resamples) to derive the optimism-corrected AUC (25). Calibration was evaluated using calibration plots (LOWESS smoothing curve), calibration intercept (ideal = 0), calibration slope (ideal = 1), the Hosmer–Lemeshow test (df = 8), and the Brier score (26).

Clinical utility was assessed using DCA (27, 28). Net benefit was compared across a threshold probability range of 0.01–0.99 for the multivariable model, BBS-only model, treat-all, and treat-none strategies. Separate DCAs were performed for ischaemic and haemorrhagic subgroups, with threshold probabilities corresponding to the subtype-specific optimal cut-offs (ischaemic 33, haemorrhagic 12) indicated on the plots.

Five sensitivity analyses were conducted to assess the robustness of the primary results. First, an NIHSS-adjusted model was compared with the base model using complete cases (*n* = 121) with available NIHSS data; Spearman correlation between BBS and NIHSS was calculated. Second, the stability of the BBS aOR was assessed across 5 models additionally adjusting for centre (dummy variables), rehabilitation length of stay, or both (Table SI). Third, to quantify the impact of heterogeneity in onset-to-admission intervals, log-transformed onset-to-admission interval was added to the base multivariable model, and independent walking rates were compared by onset-to-admission interval quartiles. Fourth, generalized estimating equations (GEE; exchangeable correlation structure) were applied with centre as the clustering unit to account for within-centre correlation. Fifth, subgroup analyses were performed for acute (Centre A) and subacute (Centres B+C) phases (Table SII), and BBS × phase interaction was tested; BBS predictive performance was examined by centre type and stroke subtype (Table SIII).

As a secondary analysis, multivariable linear regression was used to assess the association between admission BBS and discharge MBI, adjusting for admission MBI, age, sex, stroke type, and MMSE.

## RESULTS

### Participant characteristics

A total of 565 patients were included (acute university hospital 317 [56.1%]; subacute rehabilitation hospitals 248 [43.9%]). Mean age was 61.3 (SD 13.9) years, and 343 (60.7%) were male. Stroke type was ischaemic in 316 (55.9%) and haemorrhagic in 249 (44.1%). Median onset-to-admission interval was 42 days (interquartile range 22–73). Mean admission BBS was 24.2 (SD 20.3) and mean admission FAC was 1.7 (SD 1.4). NIHSS was available for 21.4% (121 patients), with a mean of 7.4 (SD 6.1).

Independent walking (FAC ≥ 4) was achieved by 214 patients (37.9%). The independent walking group had significantly higher admission BBS (*p* < 0.001), MMSE (*p* < 0.001), and FAC (*p* < 0.001), and significantly lower age (*p* < 0.001) and onset-to-admission interval (*p* < 0.001) compared with the non-independent group. Stroke type differed significantly between groups (ischaemic proportion: non-independent 53.1% vs independent 62.3%; p = 0.025). Participant characteristics are presented in Table I.

### Univariable and multivariable logistic regression

In univariable analysis, admission BBS was significantly associated with independent walking (OR = 1.100, 95% CI 1.084–1.116, *p* < 0.001). Age (OR = 0.970, *p* < 0.001), stroke type (OR = 0.663, *p* = 0.020), admission MMSE (OR = 1.078, *p* < 0.001), and admission FAC (OR = 4.129, *p* < 0.001) were also significantly associated.

In multivariable logistic regression (10 patients excluded due to missing MMSE; complete cases *n* = 555), admission BBS was a significant independent predictor of independent walking (aOR = 1.053, 95% CI 1.030–1.076, *p* < 0.001). Admission FAC (aOR = 2.822, 95% CI 2.026–3.931, *p* < 0.001) and log-transformed onset-to-admission interval (aOR = 0.430, 95% CI 0.318–0.580, *p* < 0.001) were also significant independent predictors. The McFadden pseudo-R² was 0.506. The enhanced model showed significantly improved fit compared with the base model without onset-to-admission interval (likelihood ratio test: χ² = 35.2, *p* < 0.001). Results are presented in Table II.

**Table II:** Univariable and multivariable logistic regression analysis for independent walking (FAC ≥ 4) on discharge

Variable	OR	95% CI	*p*-value	aOR	95% CI	*p*-value	
BBS T0	1.100	1.084–1.116	< 0.001	1.053	1.030–1.076	< 0.001	
Age	0.970	0.958–0.983	< 0.001	0.979	0.957–1.001	0.056	
Sex (female)	0.749	0.527–1.065	0.107	1.000	0.573–1.747	1.000	^a^
Type (haemorrhagic)	0.663	0.469–0.938	0.020	1.324	0.708–2.478	0.380	^a^
MMSE T0	1.078	1.056–1.100	< 0.001	1.005	0.973–1.038	0.759	
MBI T0	1.072	1.060–1.084	< 0.001	—	—	—	^b^
FAC T0	4.129	3.318–5.139	< 0.001	2.822	2.026–3.931	< 0.001	
BMI	1.093	1.042–1.147	< 0.001	—	—	—	
Log(Onset_T0)	—	—	—	0.430	0.318–0.580	< 0.001	^c^

Enhanced model: McFadden R² = 0.506; AIC = 379.4; LR test vs base: χ² = 35.17, *p* < 0.001; *n* = 555.

a Forced entry based on clinical relevance [4, 20].

b Excluded due to collinearity with BBS (Spearman ρ > 0.70).

c Log(Onset_T0 + 1) added to enhanced model.

aOR: adjusted odds ratio; CI: confidence interval; OR: odds ratio.

### ROC curve analysis and optimal cut-off values

BBS demonstrated excellent discriminative ability for independent walking on discharge (AUC=0.899, 95% CI 0.874–0.921). The multivariable model AUC was 0.931 (95% CI 0.911–0.950), numerically higher than the BBS-only model (0.899); however, DeLong testing within the same complete cases (*n* = 555) show-ed no statistically significant difference (Z = −1.659, *p* = 0.097). This suggests that BBS assessment alone can maintain a level of discriminative ability equivalent to the multivariable model. The optimal BBS cut-off by the Youden index was 24 points, with sensitivity 90.7%, specificity 74.4%, PPV 68.3%, and NPV 92.9%. Diagnostic performance at multiple cut-off points is presented in Table III.

**Table III:** Diagnostic performance of admission BBS at multiple cutoff points for predicting independent walking (FAC ≥ 4) on discharge

BBS cutoff	Sensitivity	Specificity	PPV	NPV	LR+	LR−
10	0.977	0.632	0.618	0.978	2.66	0.037
15	0.944	0.670	0.635	0.951	2.86	0.084
20	0.911	0.721	0.666	0.930	3.26	0.123
24*	**0.907**	**0.744**	**0.683**	**0.929**	3.54	0.126
30	0.841	0.789	0.709	0.891	3.99	0.201
33	0.790	0.832	0.741	0.866	4.70	0.253
35	0.757	0.843	0.747	0.851	4.83	0.288
40	0.673	0.897	0.800	0.818	6.56	0.364
45	0.542	0.937	0.841	0.770	8.65	0.489

Overall (*n* = 565): AUC = 0.899 (95% CI: 0.874–0.921).

Ischaemic (*n* = 316): AUC = 0.890 (95% CI: 0.851–0.924); optimal cutoff = 33.

Haemorrhagic (*n* = 249): AUC = 0.912 (95% CI: 0.872–0.945); optimal cutoff = 12.

DeLong test (ischaemic vs haemorrhagic): Z = −1.052, *p* = 0.293.

* Optimal cutoff by Youden index. PPV = positive predictive value; NPV = negative predictive value; LR = likelihood ratio.

When the ROC-derived optimal cut-off (BBS ≥ 24) was entered as a binary variable into the enhanced multivariable model, the aOR was 4.226 (95% CI 2.067–8.637, *p* < 0.001), confirming independent significance after adjustment for age, sex, stroke type, cognitive function, admission ambulatory status, and onset-to-admission interval. Subtype-specific binary analyses showed an aOR of 2.464 (95% CI 1.040–5.839, *p* = 0.040) for BBS ≥ 33 in ischaemic stroke and 24.778 (95% CI 5.491–111.80, *p* < 0.001) for BBS ≥ 12 in haemorrhagic stroke, confirming that both overall and subtype-specific cut-offs remained significant after multivariable adjustment.

### Subgroup analyses

Subtype-specific analysis. BBS demonstrated high discriminative ability for independent walking in both ischaemic and haemorrhagic stroke subtypes, although subtype-specific optimal cut-offs differed markedly. In ischaemic stroke (*n* = 316), the AUC was 0.890 (95% CI 0.851–0.924), with an optimal cut-off of 33 (sensitivity 84.1%, specificity 77.0%). In haemorrhagic stroke (*n* = 249), the AUC was 0.912 (95% CI 0.872–0.945), with an optimal cut-off of 12 (sensitivity 96.3%, specificity 75.6%).

The DeLong test showed no significant difference in AUC between subtypes (Z = −1.052, *p* = 0.293). The BBS × stroke type interaction was not significant (OR = 0.995, *p* = 0.756; likelihood ratio test χ² = 0.097, df = 1, *p* = 0.756).

*Analysis by admission ambulatory status.* BBS predictive performance varied markedly by admission ambulatory level. BBS had the highest predictive ability in the non-ambulatory group (FAC 0–1, *n* = 325; OR = 1.105, 95% CI 1.073–1.138, *p* < 0.001; AUC = 0.890), in which the independent walking rate was 10.5%. In the assisted ambulatory group (FAC 2–3, *n* = 170), the rate was 57.6% but BBS was not statistically significant (OR = 1.023, 95% CI 0.995–1.051, *p* = 0.105; AUC = 0.604). In the group already ambulatory on admission (FAC 4–5, *n* = 92), most patients already met the criterion (rate 96.7%), limiting additional discriminative contribution of BBS.

### Internal validation and calibration

Bootstrap internal validation (1,000 resamples) showed an optimism of 0.005 for the multivariable model, with an optimism-corrected AUC of 0.936 (apparent AUC 0.931). The BBS-only model optimism was −0.0003 (effectively zero), with a corrected AUC of 0.900, identical to the apparent estimate. These results indicate negligible overfitting in both models.

**Fig. 2:**
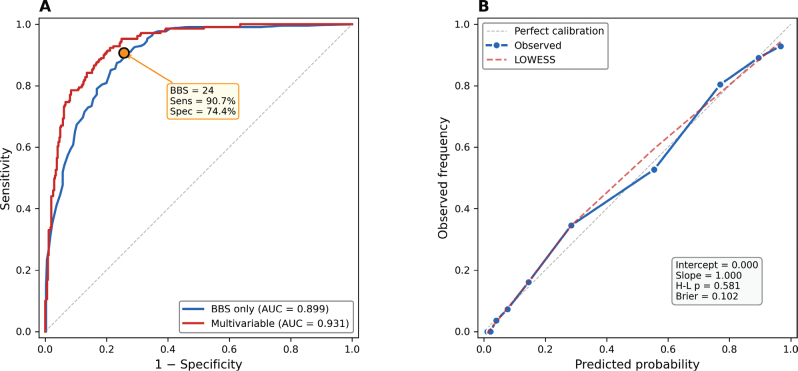
(A) Receiver operating characteristic curves for BBS-only and multivariable models. (B) Calibration plot of the multivariable prediction model.

The calibration plot showed a calibration intercept of −0.0003 (ideal 0) and slope of 0.999 (ideal 1), indicating excellent agreement between predicted and observed probabilities. The Hosmer–Lemeshow test showed no significant lack of fit (χ² = 6.595, df = 8, *p* = 0.581), and the Brier score was 0.102 (Fig. 2).

### Decision curve analysis

In DCA, both the multivariable and BBS-only models showed higher net benefit than the treat-all and treat-none strategies across the threshold probability range of 0.02–0.98. In the clinically relevant threshold range of 0.20–0.50, net benefit was 0.208–0.299 for the multivariable model and 0.189–0.296 for BBS-only; the treat-all strategy turned negative beyond a threshold of 0.40.

**Fig. 3:**
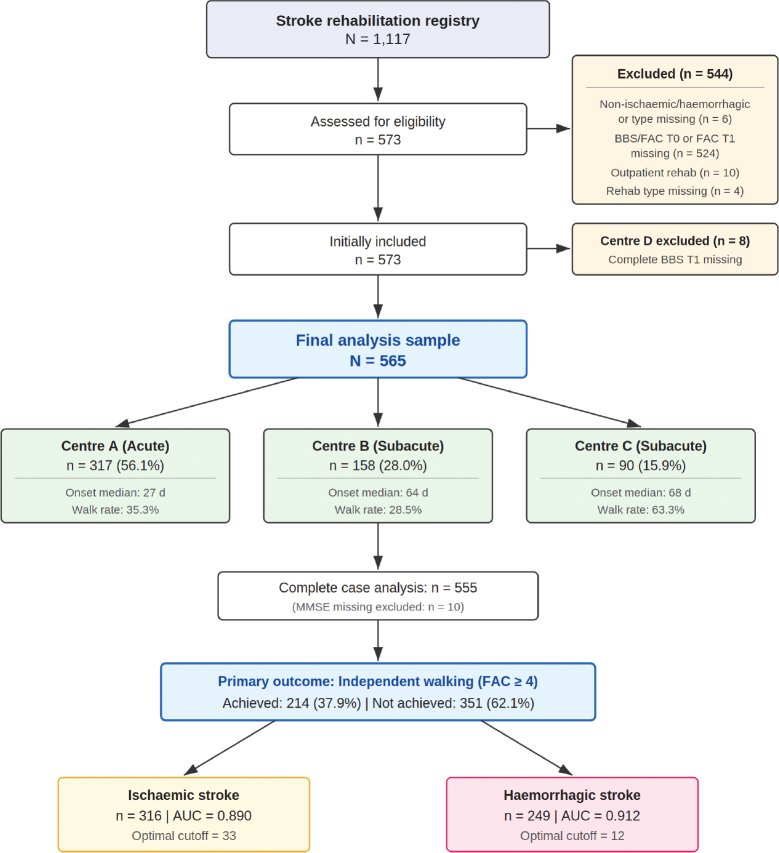
Decision curve analysis comparing BBS-only and multivariable models with treat-all and treat-none strategies.

In subtype-specific DCA, ischaemic stroke (*n* = 316) showed net benefit of 0.267 (multivariable) and 0.249 (BBS-only) at the threshold, corresponding to the 33-point cut-off (≈ 0.44). Haemorrhagic stroke (*n* = 249) showed nearly identical net benefit between models at the threshold corresponding to the 12-point cut-off (≈ 0.14). In both subtypes, the prediction models demonstrated superior net benefit compared with the treat-all strategy (Fig. 3).

### Sensitivity analyses

NIHSS-adjusted sensitivity analysis showed stable BBS predictive effects. In complete cases with available NIHSS data (*n* = 121), the BBS aOR in the base model was 1.058, and in the NIHSS-adjusted model was 1.060. A significant negative correlation was observed between admission BBS and NIHSS (Spearman ρ = −0.260, *p* = 0.004, *n* = 121).

Additional sensitivity analyses adjusting for centre and rehabilitation length of stay also showed stable BBS predictive effects. The BBS aOR was 1.056 (95% CI 1.033–1.080) in the centre-adjusted model, 1.056 (95% CI 1.034–1.079) in the log(rehabilitation duration)-adjusted model, and 1.050 (95% CI 1.026–1.075) in the maximally adjusted model including centre, rehabilitation duration, and onset-to-admission interval. When admission FAC was excluded from the model, the BBS aOR increased to 1.108 (95% CI 1.088–1.128), consistent with expected attenuation from collinearity in the primary model. Across all models, the BBS aOR remained within the range 1.046–1.108 (see Table SI). Leave-one-centre-out analysis showed BBS aOR ranging from 1.029 to 1.066 regardless of which centre was excluded. GEE analysis accounting for within-centre clustering yielded a BBS aOR of 1.046 (95% CI 1.023–1.069, *p* < 0.001), with low intraclass correlation (ICC = 0.077), confirming that clustering had no material impact on the results.

Subgroup analysis of acute (Centre A, *n* = 317) and subacute (Centres B+C, *n* = 248) phases showed BBS aOR of 1.046 (95% CI 1.014–1.078) and 1.070 (95% CI 1.034–1.107), respectively, both significant. BBS-only AUC was 0.922 (acute) and 0.883 (subacute), with no statistical difference (bootstrap ΔAUC *p* = 0.126), but optimal cut-offs differed markedly: acute 10 (sensitivity 97.3%, specificity 77.6%) vs subacute 38 (sensitivity 81.4%, specificity 79.5%), a difference of 28 points. The BBS × rehabilitation phase interaction was not significant (likelihood ratio test: χ² = 0.012, *p* = 0.912).

### Secondary outcome analysis

In multivariable linear regression, admission BBS was significantly positively associated with discharge MBI (β = 0.732, 95% CI 0.646–0.818, *p* < 0.001). A significant positive correlation was found between admission BBS and FAC change (Spearman ρ = 0.084, *p* = 0.042), while the correlation with MBI change was not significant (ρ = −0.030, *p* = 0.478).

## DISCUSSION

This multicentre retrospective cohort study demonstrated that admission BBS score is an independent predictor of independent walking on discharge in inpatient stroke rehabilitation patients. The most important finding was that optimal BBS cut-offs for independent walking were 33 for ischaemic stroke and 12 for haemorrhagic stroke, revealing a 21-point dissociation between subtypes. This was established for the first time through direct statistical comparison within the same cohort. These results suggest limitations in applying a single cut-off value for walking prognosis prediction and strongly support the clinical need for subtype-specific precision rehabilitation decision-making.

BBS demonstrated high discriminative ability for predicting independent walking after stroke. The AUC of 0.899 in the present study exceeds the previously reported range of 0.73–0.88 for BBS walking prediction (7, 29, 30, 31). This may be explained by several factors: the clinical homogeneity of restricting the study population to inpatient rehabilitation patients, the minimization of outcome measurement error through dichotomisation of walking outcome using the clear FAC ≥ 4 criterion (32), and the enhanced representativeness achieved through multicentre sampling while controlling for single-centre biases.

The BBS-only model showed statistically equivalent discriminative ability to the multivariable model. Within the same complete cases (*n* = 555), the DeLong test showed no significant difference in AUC between the two models (Z = −1.659, *p* = 0.097). This indicates that adding multiple covariates including age, sex, and stroke type does not achieve a meaningful improvement in discriminative ability over the BBS-only model. In the clinical context of early inpatient rehabilitation, where rapid prognostic assessment directly informs treatment planning and resource allocation, the finding that a single standardized assessment tool provides sufficient predictive ability carries important practical value in itself.

A BBS score of 24 was identified as a clinical reference cut-off with high reliability for early identification of patients unlikely to achieve independent walking. This cut-off showed sensitivity of 90.7%, specificity of 74.4%, and NPV of 92.9%, meaning that 92.9% of patients scoring below 24 did not achieve independent walking on discharge. Previous studies have reported BBS cut-offs ranging from 20 to 45 for predicting walking after stroke (33), and this heterogeneity is likely attributable to differences in study population composition, outcome definitions, and assessment timing. The high NPV of the present study provides practical evidence for realistic prognostic counselling and proactive planning of complementary mobility strategies during early rehabilitation.

The 21-point cut-off dissociation between ischaemic and haemorrhagic stroke directly reflects mechanistic differences in functional recovery between the 2 subtypes. In haemorrhagic stroke, the acute haematoma and surrounding perihematomal oedema temporarily and reversibly suppress neural function, causing initial BBS scores to be lower than the actual extent of neural damage. As haematoma absorption and oedema resolution progress, reversible recovery occurs, allowing independent walking to be achieved even from relatively low initial BBS scores (13, 16). In contrast, functional recovery in ischaemic stroke depends on the structural integrity of the corticospinal tract and neuroplasticity (12), and initial balance ability more directly reflects the irreversible extent of damage, resulting in a higher BBS threshold for achieving independent walking. The homogeneity of AUC between subtypes (DeLong: Z = −1.052, *p* = 0.293) and the non-significance of the interaction test (LRT *p* = 0.756) statistically confirm that the predictive mechanism of BBS is equally valid in both subtypes despite the numerical difference in cut-off values, supporting the methodological validity of applying subtype-specific cut-offs. Notably, the binary variable analysis for haemorrhagic stroke yielded a large aOR (24.778) with a wide confidence interval (5.491–111.80), attributable to quasi-complete separation in which only 3 of 130 patients with BBS < 12 achieved independent walking. While the lower bound of the confidence interval (5.491) exceeds unity, confirming statistical significance, the precise magnitude of the estimate should be regarded as exploratory and requires validation in larger haemorrhagic stroke cohorts.

The BBS-only model demonstrated reliability not only in discrimination but also in calibration and clinical decision-making. Bootstrap internal validation showed a corrected AUC of 0.900, with a difference of only 0.005 from the original value (0.899), indicating extremely low overfitting potential (25). Calibration analysis showed an intercept of −0.0003 and slope of 0.999, closely approximating perfect calibration, with satisfactory fit confirmed by the Hosmer–Lemeshow test (*p* = 0.581) and Brier score (0.102). In DCA, the BBS-only model showed positive net benefit across the wide threshold range of 0.02–0.98, with a clear clinical advantage over the treat-all strategy beyond a threshold of 0.40 (27). This indicates that BBS-based model judgement provides superior net benefit compared with intuitive clinical judgement in situations where the probability of achieving independent walking is judged to be 40% or higher.

Sensitivity analysis confirmed that BBS provides independent predictive information distinct from neurological severity. The BBS aOR changed only from 1.058 to 1.060 after additional NIHSS adjustment (change < 0.2%), and the Spearman correlation between BBS and NIHSS (ρ = −0.260, *p* = 0.004) indicated statistical association without substantial multicollinearity. BBS thus appears to capture not only the degree of neurological impairment but also the actual functional balance performance capacity, a functional compensatory capacity that varies between individuals even when the degree of neurological damage is equivalent. However, NIHSS data were available for only 21.4% (*n* = 121) of the cohort, primarily due to incomplete electronic medical records in the retrospective data collection, limiting the representativeness of this sensitivity analysis. Systematic NIHSS collection is recommended in future prospective studies.

The multicentre composition of this study provides an important methodological strength for verifying the stability of the BBS prediction model in real clinical environments. The 3 participating centres comprised 1 acute university hospital (Centre A; median length of stay 47 days) and 2 subacute rehabilitation hospitals (Centre B 201 days, Centre C 180 days), comprehensively representing the rehabilitation delivery system from early post-stroke to long-term rehabilitation. Even in the maximally adjusted model including centre, rehabilitation duration, and onset-to-admission interval, the BBS aOR was stable at 1.050 (95% CI 1.026–1.075), and leave-one-centre-out analysis showed stable BBS aOR in the range 1.029–1.066 regardless of which centre was excluded. This supports the generalizable applicability of the BBS prediction model across diverse rehabilitation healthcare systems rather than being specific to a single institutional environment.

Subgroup analysis of acute and subacute phases provided important exploratory findings regarding the phase-specific application of the BBS prediction model. While BBS discriminative ability (AUC) was statistically homogeneous between groups (acute 0.922 vs subacute 0.883; bootstrap *p* = 0.126), optimal cut-offs differed by 28 points (acute 10 vs subacute 38). The low cut-off in acute patients may be explained by 2 mechanisms: first, rapid functional recovery expected during the early neuroplasticity window allows independent walking from low baseline balance; second, the daily BBS change rate in acute patients (0.303 points/day) was approximately 7-fold higher than in subacute patients (0.045 points/day). In subacute patients, the functional recovery trajectory has reached a plateau, requiring relatively high baseline balance (BBS ≥ 38) for independent walking. However, as the acute group comprised a single centre (Centre A), centre and phase effects are confounded, and as the BBS × phase interaction was not significant (*p* = 0.912), this cut-off difference should be interpreted as an exploratory finding requiring independent verification through future prospective studies with within-centre phase control.

The findings of this study provide specific implications for clinical practice in rehabilitation medicine. Using a BBS cut-off of 24 with an NPV of 92.9%, patient-specific walking training strategies can be established early in rehabilitation. For patients scoring 24 or above, early intensive gait training can be actively initiated. For those scoring below 24, alternative walking approaches such as functional electrical stimulation (FES), robot-assisted gait training, and bodyweight-supported treadmill training can be introduced early, with individualized intermediate goals set for stepwise improvement of balance ability. This represents not the exclusion of patients below a certain score from walking training, but a clinical reference point for designing rehabilitation pathways optimized to each patient’s current functional level. Application of subtype-specific cut-offs (ischaemic 33, haemorrhagic 12) enhances precision in goal-setting and prognostic counselling. As the BBS-only model showed equivalent clinical predictive ability to the multivariable model, immediate treatment planning using a single standardized assessment tool is feasible even in resource-limited clinical settings.

### Limitations

This study has the following limitations. First, the retrospective cohort design cannot fully exclude residual confounding by unmeasured variables such as lesion location, lesion volume, and rehabilitation intensity. Second, only 565 of 1,117 registry patients (50.6%) were included, with the primary exclusion reason being missing BBS data (58.5%), which may have been more frequent in more severely affected patients (comparison of included and excluded patients is presented in Table SIV). Third, only internal validation was performed; external validation using an independent cohort was not conducted. As this study is based on a single-country cohort, additional studies are needed to establish the racial and geographical generalizability of the derived cut-offs. Fourth, 1 of the 3 centres (Centre A) accounted for 56.1% of the total sample, creating centre-level sample imbalance, and inter-rater reliability could not be directly verified due to the retrospective design. Fifth, rehabilitation length of stay at Centres B and C (median 201 and 180 days, respectively) reflects centre-specific inpatient duration and may include patients transferred from other facilities, consistent with the Korean subacute rehabilitation pathway; this heterogeneity in admission route was not systematically recorded and may limit the comparability of this variable across centres.

### Conclusion

This multicentre retrospective cohort study demonstrated that admission BBS score is an independent predictor of independent walking on discharge in inpatient stroke rehabilitation patients (aOR = 1.053, 95% CI 1.030–1.076; AUC = 0.899). The enhanced model with additional onset-to-admission interval adjustment (McFadden R² = 0.506) showed significantly improved fit over the base model, and BBS predictive effects were stable across all sensitivity analyses (aOR range 1.046–1.070). The finding that the difference in discriminative ability from the multivariable model was not statistically significant (DeLong *p* = 0.097) directly supports the conclusion that equivalent prognostic predictive ability can be maintained using BBS assessment alone without additional covariate collection, underscoring its practical value as a single standardized tool. Most importantly, direct statistical comparison within the same cohort revealed for the first time a 21-point dissociation in optimal cut-off values between ischaemic (33) and haemorrhagic (12) stroke, reflecting mechanistic differences in functional recovery between subtypes and providing concrete clinical evidence for subtype-specific precision rehabilitation strategies. To establish the universal applicability of these subtype-specific cut-offs, external validation using multinational independent cohorts with racial and geographical diversity is warranted.

## References

[1] Feigin VL, Owolabi MO, World Stroke Organization–Lancet Neurology Commission Stroke Collaboration Group. World Stroke Organization (WSO): Global Stroke Fact Sheet 2025. Int J Stroke 2025. 10.1177/17474930241301355

[2] Jørgensen HS, Nakayama H, Raaschou HO, Olsen TS. Recovery of walking function in stroke patients: the Copenhagen Stroke Study. Arch Phys Med Rehabil 1995; 76: 27–32. 10.1016/S0003-9993(95)80038-7 7811170

[3] Bohannon RW, Andrews AW, Smith MB. Rehabilitation goals of patients with hemiplegia. Int J Rehabil Res 1988; 11: 181–184.

[4] Kwakkel G, Wagenaar RC, Kollen BJ, Lankhorst GJ. Predicting disability in stroke: a critical review of the literature. Age Ageing 1996; 25: 479–489. 10.1093/ageing/25.6.479 9003886

[5] Blum L, Korner-Bitensky N. Usefulness of the Berg Balance Scale in stroke rehabilitation: a systematic review. Phys Ther 2008; 88: 559–566. 10.2522/ptj.20070205 18292215

[6] Mao HF, Hsueh IP, Tang PF, Sheu CF, Hsieh CL. Analysis and comparison of the psychometric properties of three balance measures for stroke patients. Stroke 2002; 33: 1022–1027. 10.1161/01.STR.0000012516.63191.C5 11935055

[7] Louie DR, Eng JJ. Berg Balance Scale score at admission can predict walking suitable for community ambulation at discharge from inpatient stroke rehabilitation. J Rehabil Med 2018; 50: 37–44. 10.2340/16501977-2280 29068037

[8] Veerbeek JM, Kwakkel G, van Wegen EE, Ket JC, Heymans MW. Early prediction of outcome of activities of daily living after stroke: a systematic review. Stroke 2011; 42: 1482–1488. 10.1161/STROKEAHA.110.604090 21474812

[9] Veerbeek JM, Van Wegen EE, Harmeling-Van der Wel BC, Kwakkel G; EPOS Investigators. Is accurate prediction of gait in nonambulatory stroke patients possible within 72 hours poststroke? The EPOS study. Neurorehabil Neural Repair 2011; 25: 268–274. 10.1177/1545968310384271 21186329

[10] Wouda NC, Knijff B, Punt M, Visser-Meily JMA, Pisters MF. Predicting recovery of independent walking after stroke: a systematic review. Am J Phys Med Rehabil 2024; 103: 458–464. 10.1097/PHM.0000000000002436 38363655

[11] Stinear CM, Barber PA, Petoe M, Anber S, Byblow WD. Prediction of motor recovery after stroke: advances in biomarkers. Lancet Neurol 2017; 16: 826–836. 10.1016/S1474-4422(17)30283-1 28920888

[12] Kitago T, Ratan RR. Rehabilitation following hemorrhagic stroke: building the case for stroke-subtype specific recovery therapies. F1000Research 2017; 6: 2044. 10.12688/f1000research.11913.1 29250322 PMC5701438

[13] Paolucci S, Antonucci G, Grasso MG, Bragoni M, Coiro P, De Angelis D, et al. Functional outcome of ischemic and hemorrhagic stroke patients after inpatient rehabilitation: a matched comparison. Stroke 2003; 34: 2861–2865. 10.1161/01.STR.0000102902.39759.D3 14615613

[14] Kelly PJ, Furie KL, Shafqat S, Rallis N, Chang Y, Stein J. Functional recovery following rehabilitation after hemorrhagic and ischemic stroke. Arch Phys Med Rehabil 2003; 84: 968–972. 10.1016/S0003-9993(03)00040-6 12881818

[15] Chu CL, Chen YP, Chen CCP, Chen CK, Chang HN, Chang CH, et al. Functional recovery patterns of hemorrhagic and ischemic stroke patients under post-acute care rehabilitation program. Neuropsychiatr Dis Treat 2020; 16: 1975–1985. 10.2147/NDT.S253352 32884273 PMC7431596

[16] Perna R, Temple J. Rehabilitation outcomes: ischemic versus hemorrhagic strokes. Behav Neurol 2015; 2015: 891651. 10.1155/2015/891651 26246694 PMC4515256

[17] von Elm E, Altman DG, Egger M, Pocock SJ, Gøtzsche PC, Vandenbroucke JP. The Strengthening the Reporting of Observational Studies in Epidemiology (STROBE) statement. Lancet 2007; 370: 1453–1457. 10.1016/S0140-6736(07)61602-X 18064739

[18] Collins GS, Reitsma JB, Altman DG, Moons KG. Transparent Reporting of a multivariable prediction model for Individual Prognosis or Diagnosis (TRIPOD): the TRIPOD statement. Ann Intern Med 2015; 162: 55–63. 10.7326/M14-0697 25560714

[19] Berg KO, Wood-Dauphinee SL, Williams JI, Maki B. Measuring balance in the elderly: validation of an instrument. Can J Public Health 1992; 83 Suppl 2: S7–11. 1468055

[20] Preston E, Ada L, Stanton R, Mahendran N, Dean CM. Prediction of independent walking in people who are nonambulatory early after stroke: a systematic review. Stroke 2021; 52: 3217–3224. 10.1161/STROKEAHA.120.032345 34238016

[21] Folstein MF, Folstein SE, McHugh PR. “Mini-mental state”: a practical method for grading the cognitive state of patients for the clinician. J Psychiatr Res 1975; 12: 189–198. 10.1016/0022-3956(75)90026-6 1202204

[22] Shah S, Vanclay F, Cooper B. Improving the sensitivity of the Barthel Index for stroke rehabilitation. J Clin Epidemiol 1989; 42: 703–709. 10.1016/0895-4356(89)90065-6 2760661

[23] Holden MK, Gill KM, Magliozzi MR, Nathan J, Piehl-Baker L. Clinical gait assessment in the neurologically impaired: reliability and meaningfulness. Phys Ther 1984; 64: 35–40. 10.1093/ptj/64.1.35 6691052

[24] DeLong ER, DeLong DM, Clarke-Pearson DL. Comparing the areas under two or more correlated receiver operating characteristic curves: a nonparametric approach. Biometrics 1988; 44: 837–845. 3203132

[25] Steyerberg EW, Harrell FE Jr, Borsboom GJ, Eijkemans MJ, Vergouwe Y, Habbema JD. Internal validation of predictive models: efficiency of some procedures for logistic regression analysis. J Clin Epidemiol 2001; 54: 774–781. 10.1016/S0895-4356(01)00341-9 11470385

[26] Hosmer DW, Lemeshow S, Sturdivant RX. Applied logistic regression. 3rd ed. Hoboken, NJ: Wiley; 2013.

[27] Vickers AJ, Elkin EB. Decision curve analysis: a novel method for evaluating prediction models. Med Decis Making 2006; 26: 565–574. 10.1177/0272989X06295361 17099194 PMC2577036

[28] Vickers AJ, van Calster B, Steyerberg EW. A simple, step-by-step guide to interpreting decision curve analysis. Diagn Progn Res 2019; 3: 18. 10.1186/s41512-019-0064-7 31592444 PMC6777022

[29] Liao WL, Chang CW, Sung PY, Hsu WN, Lai MW, Tsai SW. The Berg Balance Scale at admission can predict community ambulation at discharge in patients with stroke. Medicina 2021; 57: 556. 10.3390/medicina57060556 34072817 PMC8226946

[30] Makizako H, Kabe N, Takano A, Isobe K. Use of the Berg Balance Scale to predict independent gait after stroke: a study of an inpatient population in Japan. PM R 2015; 7: 471–478. 10.1016/j.pmrj.2015.01.015 25633633

[31] Jenkin J, Parkinson S, Jacques A, Kho L, Hill K. Berg Balance Scale score as a predictor of independent walking at discharge among adult stroke survivors. Physiother Can 2021; 73: 252–256. 10.3138/ptc-2019-0103 34456442 PMC8370698

[32] Mehrholz J, Wagner K, Rutte K, Meissner D, Pohl M. Predictive validity and responsiveness of the functional ambulation category in hemiparetic patients after stroke. Arch Phys Med Rehabil 2007; 88: 1314–1319. 10.1016/j.apmr.2007.06.764 17908575

[33] Tyson SF, Connell LA. How to measure balance in clinical practice: a systematic review of the psychometrics and clinical utility of measures of balance activity for neurological conditions. Clin Rehabil 2009; 23: 824–840. 10.1177/0269215509335018 19656816

